# High prevalence of persistently infected animals from bovine viral diarrhea in Colombian cattle

**DOI:** 10.1186/s12917-018-1769-5

**Published:** 2019-01-10

**Authors:** Juan Quintero Barbosa, Adriana P. Corredor Figueroa, Sandra S. Salas, Hugo Camargo, Alfredo Sanchéz, Julio Tobón, Diego Ortiz, Eric Schachtebeck, Maria Fernanda Gutierrez

**Affiliations:** 1 Escuela de Biología, Universidad Industrial de Santander, Carrera 27 Calle 9, ed 45, Bucaramanga, Colombia; 2 Departamento de Microbiología, Pontificia Universidad Javeriana, Carrera 7 No. 40 – 62, Bogotá, Colombia; 3 Empresa Colombiana de Productos Veterinarios – VECOL, Av. Eldorado 82 -93, Bogotá, Colombia; 4 Corporación Colombiana de Investigación Agropecuaria – CORPOICA, Km 14 Vía Mosquera-Bogotá, Mosquera, Colombia; 5 Facultad de Veterinaria, Universidad Antonio Nariño, Carrera 3 Este # 47 A – 15, Bogotá, Colombia

**Keywords:** Bovine viral diarrhea virus, Persistently infected animals, Genotype

## Abstract

**Background:**

Bovine Viral Diarrhea Virus (BVDV) is associated with gastrointestinal, respiratory and reproductive diseases of livestock across the world that causes continuous economic losses in the cattle industry. This virus can establish a persistent infection (PI) in calves after the fetal infection, making BVDV positive catle carriers and primary reservoirs which will constantly transmit the virus to healthy and new-born animals. For this reason, the detection of the PI animals in herds is the first line of prevention of the viral infection.

**Results:**

In this study, PI animals were detected in five different regions of Colombia through RT-PCR techniques and confirmed by sequencing. BVDV genotypes were determined using one fragment of the 5’UTR. It was found a 7% BVDV prevalence in animals and 22% in farms; and genotype 1 was identified as a single genotype for all of the samples. All samples were BVDV 1a.

**Conclusion:**

This is the first report in Colombia with higher prevalence rates compared with other places in the world, turned out to be of great importance for the ranchers, the vaccine producers and animal health control parties.

## Background

Bovine Viral Diarrhea Virus (BVDV) belongs to the genus *Pestiviru*s, within the family *Flaviviridae.* Based on viral RNA sequence, there are eleven species, two of them are BVDV 1 which is the same *Pestivirus* A and BVDV 2 which is the same *Pestivirus* B, both infecting cattle [1–3]. As well as the viruses of the border disease in sheep (*Pestivirus* D) and the classical swine fever in swine (*Pestivirus* C), the BVDV is able to infect a large number of different animal species [3]. This virus is actually classified in species, genotypes and biotypes. The genotypes are mainly determined by the changes in the 5’UTR region, Npro and in the E2 protein-coding region. There are at least 20 genotypes within the BVDV- 1 which is the most common and the less frequent one called BVDV-2 has 4 genotypes more. Recently, HoBi-like pestivirus has been proposed as a new species of the genus based on its genetic and antigenic closeness to BVDV-1 and BVDV-2 [4–6]. Furthermore, isolates of BVDV can be separated into non-cytopathogenic and cytopathogenic biotypes based on cytopathogenic effects observed in infected cell cultures [4, 7] or for the heterologous recombination between non cytopathic virus and cellular sequences [4, 8].

The BVDV is one of the principal causal agents of respiratory disorders and reproductive diseases including abortions in cattle. The infection by this virus could causes different symptoms in the animals and also the development of the disease in different ways. Usually, the viral infection could be asymptomatic or with few clinical signs like mild fever, lack of appetite, lethargy but in some animals could be involved also with ulcers in the mouth and in the digestive tract, induce respiratory and reproductive implications as well as immunosuppression, in few cases could present a classic or hemorrhagic diarrhea [7, 9].

The second manifestation of the disease, is associated with the transmission of the virus through the placenta from the cow to the calf, depending on the stages of pregnancy, several outcomes could occur including embryonic death, abortion, immunotolerance, birth defects, birth of weak calves although in some cases normal calves could also be born with protective antibodies [7]. PI calves are the result of an in utero infection during the first 120 days of gestation with a noncytopathic biotype of BVDV. Such calves are born immunotolerant to the infecting viral strain and will sustain viral replication and excretion for the rest of their lives. They are consistently viremic, and constantly shed live virus in all bodily secretions. [1, 9] The creation of PI animals only follows infection with viral strains of the noncytopathic biotype, the predominant field biotype [10]. Additionally, all calves born to PI dams will be PI themselves. Many PI animals die within the first year of age; however, others can appear normal and thus serve as the viral reservoir and represent a continuous threat to herd mates and other livestock contacts [10, 11].

In PI animals thrombocytopenic signs are associated with the mucosal membranes and could be seen as hemorrhagic processes with pyrexia, anorexia, acute diarrhea and ulcers in the oral cavity; high mortality rates have been reported under these conditions [4].

BVDV is distributed worldwide and its seroprevalence has been reported between 40 and 80% taking into account the acute infection of the virus; on the other side, PI animals’ prevalence is between 0.5 and 4% although some other studies around the world are needed. Particularly in Colombia there are few studies directed to this virus; in 1996 it was described the presence of PI animals by means of lymphocytes’ cultures obtained from dairy cattle from the Savannah of Bogota [12]. In a recent study conducted by Ortiz *et. al* in 2016, it was found a seroprevalence of antibodies against the virus in the 32% of the animals and 83% in farms (submitted data). However, the current status of the PI animals is still unknown in the country even though it is essential to know the presence of these animals since it is the first step for the elimination and eradication of the virus in a specific region. For this reason, the aim of this study was to determine the prevalence of PI animals in some regions of Colombia as well as to identify the circulating species in this population, in order to establish a baseline with respect to the presence of the virus in Colombian cattle.

## Results

### RT-PCR sensitivity test

**Fig. 1:**
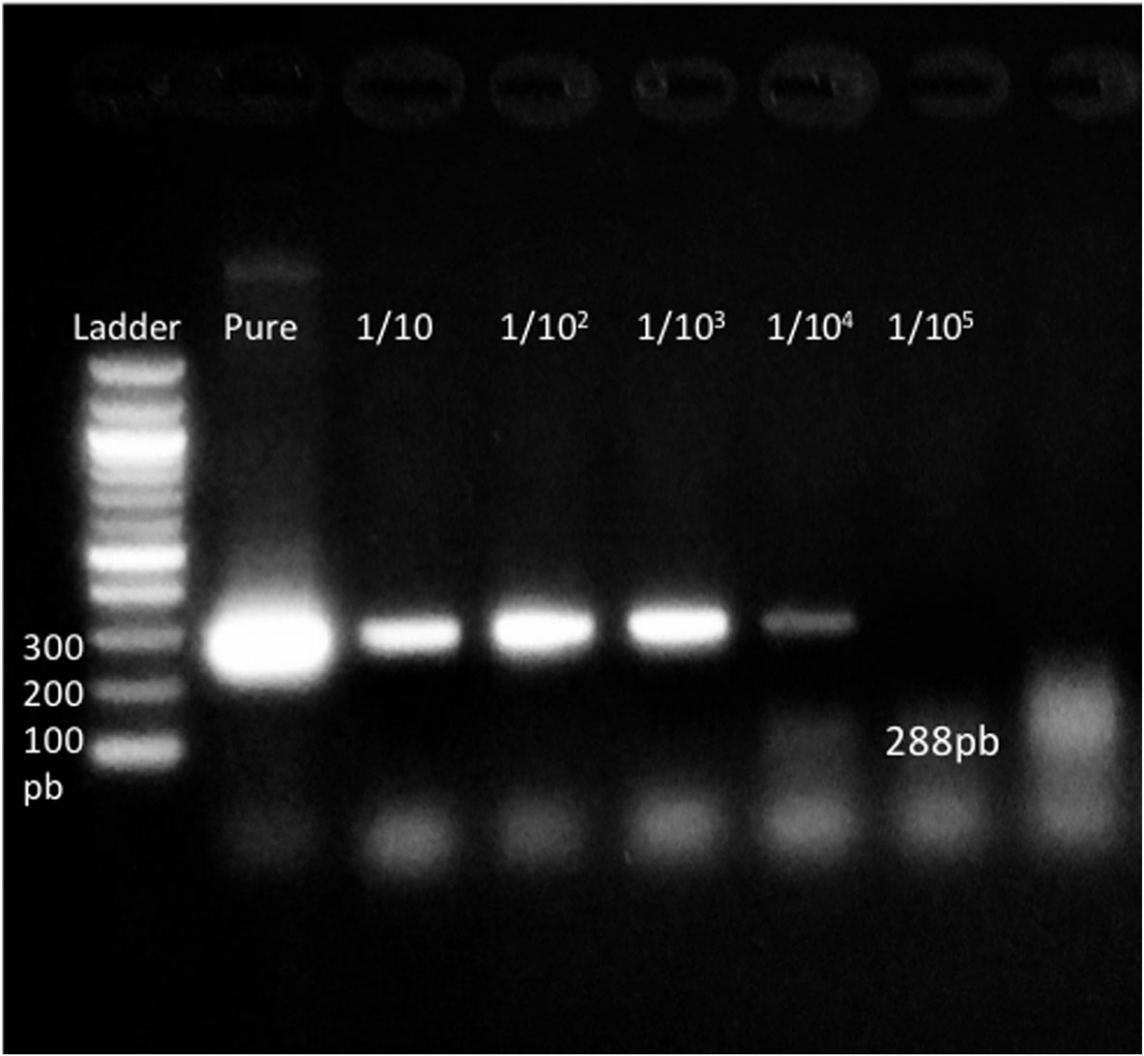
Sensitivity test of the RT-PCR using the purified plasmid pELMO for the detection of the region 5’UTR. Lane 1–100 bp ladder, lane 2 – Purified plasmid product, lane 3–7 – 10-base serial dilutions of the purified plasmid (10^-1^-10^-5^). Maximum dilution visualized with this PCR could be seen in lane 6 (10^-4^), belonging to a minimum concentration of 0.00539 pM

In the sensitivity test of the RT-PCR it was possible to amplify the purified plasmid with the viral segment in it until the dilution of 10^− 4^, which corresponds to a concentration of 0,00539 pM. In the Fig. 1 could be seen the results of the serial dilutions compared with the purified product of the plasmid.

### Prevalence of BVDV persistently infected (PI) animals

**Fig. 2:**
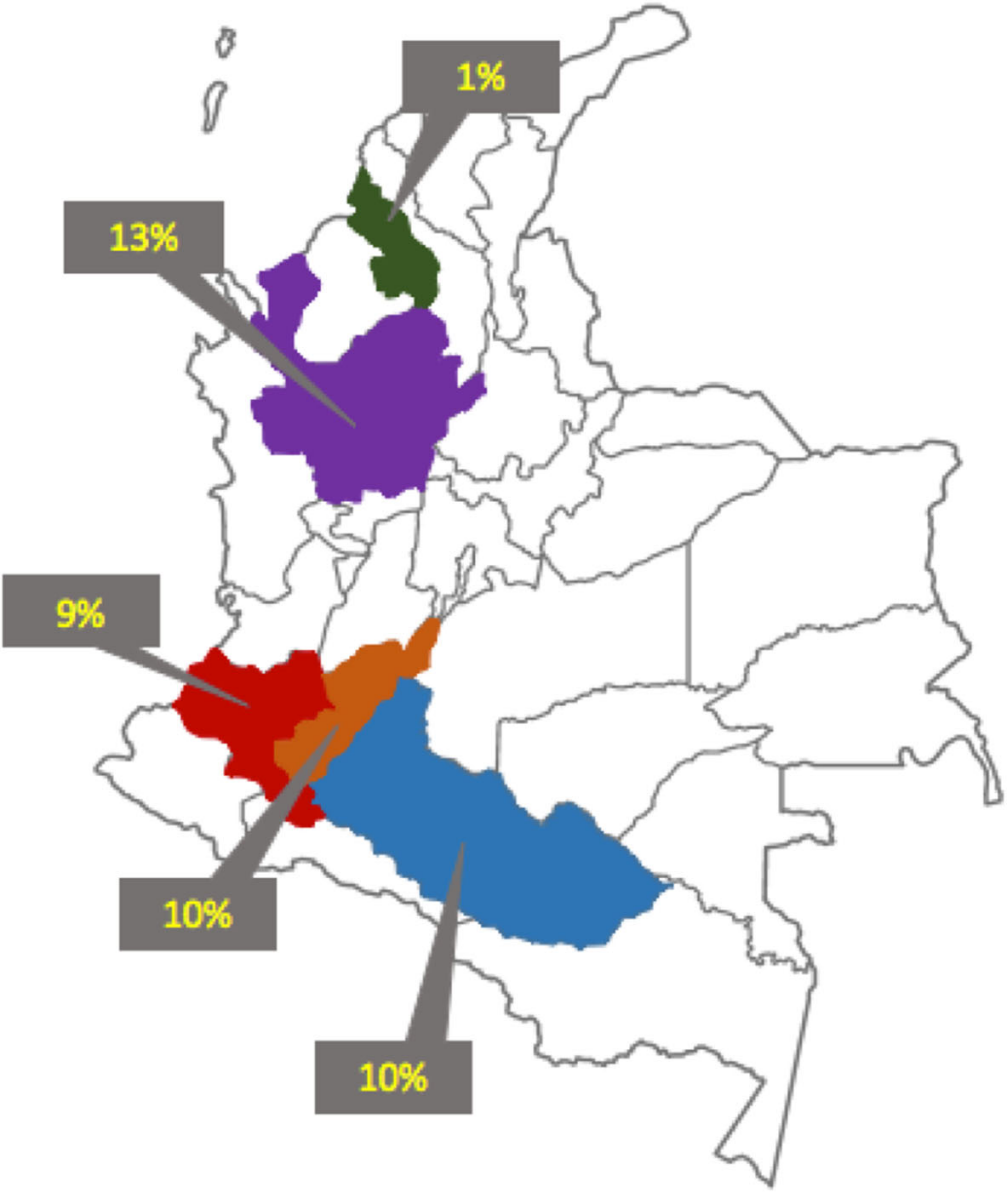
Distribution of BVDV persistently infected animals in Colombia. Green – Sucre, Purple – Antioquia, Red – Cauca, Orange – Huila, Blue – Caquetá

In the present study, a total of 260 ear notch biopsies were included for the BVDV detection from seronegative cattle collected from 45 farms located in 5 regions of Colombia. A global prevalence of 7% (19/260) was found in the studied population distributed in the country as follows: 10% (4/40) in Caquetá, 13% (7/53) in Antioquia, 9% (4/47) in Cauca, 10% (3/30) in Huila and 1% (1/90) in Sucre. Figure 2 shows the location and the prevalence percentages of PI animals in the different departments of Colombia. At the farm level, it was considered a positive farm if one or more animals were found positive in the PCR test. Thus, from the 45 analyzed farms, 10 of them were found positive to persistently infected animals corresponding to the 22% of all of the properties. The distribution of the positive farms in the 5 regions was: 3 of 5 (60%) in Caquetá, 1 of 3 (33%) in Antioquia, 4 of 20 (20%) in Cauca, 1 of 3 (33%) in Huila and 1 of 14 parcels (7%) in Sucre.

### BVDV species and subspecies

**Fig. 3:**
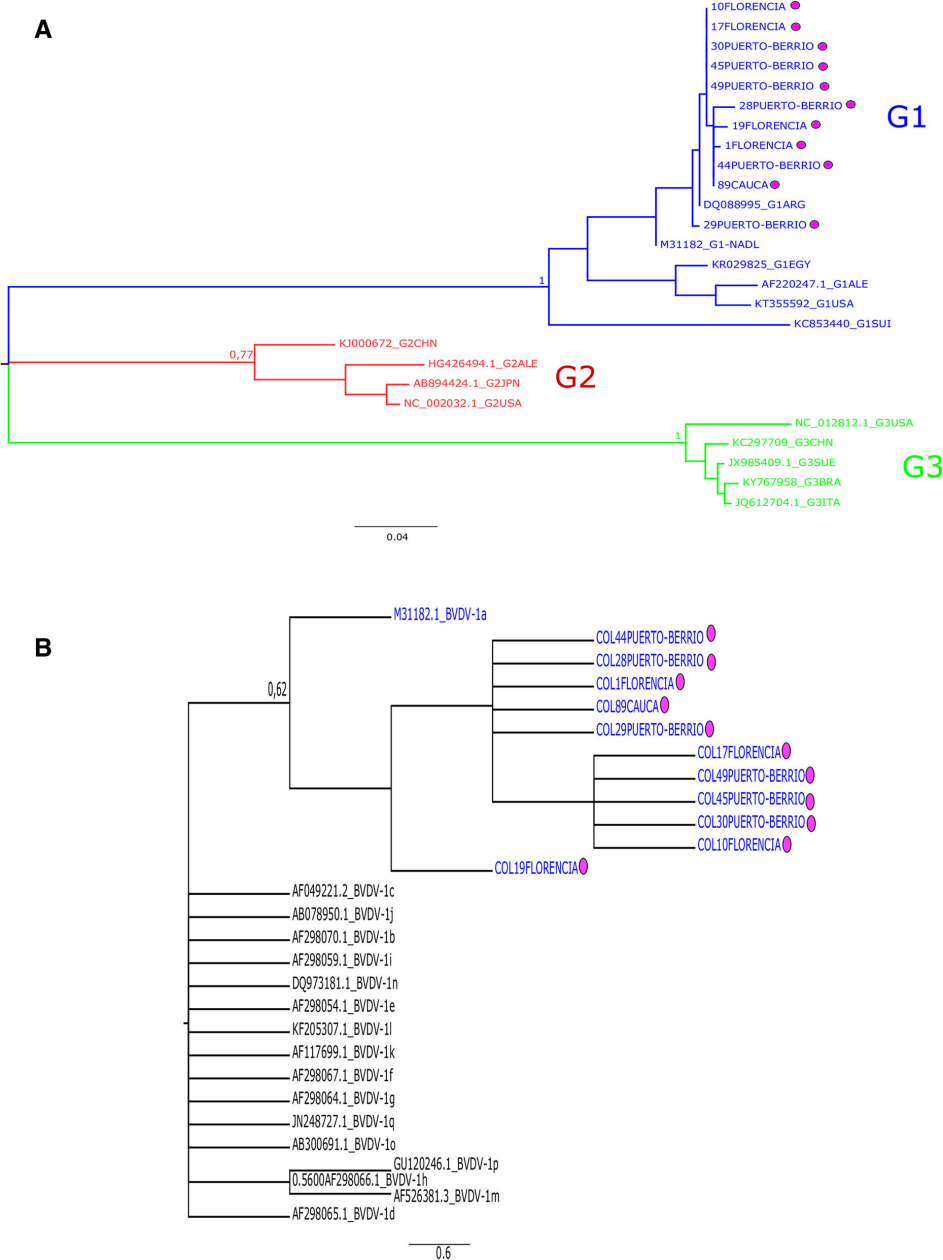
Phylogenetic reconstruction of the 5’UTR of BVDV isolated of PI animals. Only 11/19 strains could be sequenced because of the quality of the DNA **a**) Maximum Likelihood (ML) phylogenetic analysis. The evolutionary history was derived using the method of ML based on the model of General Time-Reversible + Gamma Model. The analysis was performed with 26 nucleotide sequences and a total of 325 positions in the data set. Colombian isolates were grouped in the genotype 1 (Blue), (labeled with circles in pink) with a bootstrap of 1. **b** Sub genotyping the reference strains of BVDV 1 (from *a* to *q*) to assemble the tree were selected from the data set of the GenBank. Their accession numbers are included in the tree. These sequences were aligned with the 11 Colombian sequences. The evolutionary history was derived using the method of ML based on the model of General Time-Reversible Model GAMMA and validated using bootstrap analysis with 100 replicates. The analysis involved 28 nucleotide sequences. There were a total of 232 positions in the final dataset. Colombian isolates were grouped in the Subtype 1a (Blue), (labeled with circles in pink) with a bootstrap of 0.62

In the studied population, the results obtained from the ML phylogenetic tree show that the circulating genotype of BVDV from the Colombian isolates belong to BVDV-1. In the phylogenetic tree (Fig. 3a) could be seen three different clades that represent each genotype, in which Colombian isolates obtained from PI animals are located in the BVDV-1 clade, with a bootstrap value of 100. On the other hand, with the results obtained from the likelihood mapping, it was found that the well-resolved tree was about 80.3%. This analysis statistically supports the robustness of the selected genomic region (Fig. 3a).

For genotypes, the reference strains of BVDV-1 (from a to q) to assemble the tree were selected from the data set of the GenBank. Their accession numbers are included in the tree. These sequences were aligned with the 11 Colombian sequences using MUSCLE. Phylogenetic tree was constructed using the ML method and validated using bootstrap analysis with 100 replicates. For genotypes, all the Colombian strains were BVDV species G1 genotype *a* (Fig. 3b) [13, 14].

## Discussion

The bovine viral diarrhea is one of the 10 infections in cattle with a highest impact in the animal health [15]. Even if the availability of the vaccine has been considered as one of the main prevention strategies for the viral infection, with the purpose of decreasing the incidence and prevalence rates, this strategy has not been successful at all as high rates of seroprevalence against BVDV are still found worldwide [16]. Thus, establishing the risk factors for the viral infection is a good strategy in order to avoid the viral spread, mainly in the acute infections. However, this strategy is neither enough because it does not prevent the viral spread from the PI animals, due to the fact that these animals excrete huge amounts of viral particles during the course of their lives, and is now considered the main cause of the viral dissemination.

Therefore, the identification and elimination of PI animals in conjunction with the implementation of Good Veterinary Practices as well as biosecurity conditions, are crucial for the control and prevention of the disease [4]. Eradication programs have been successfully carried out in the Scandinavian countries through the implementation of three main practices: identification, elimination and constant monitoring of PI animals in the herds [17].

In pursuance of identifying PI animals in the herds, viral isolations or RT-PCR could be done as diagnostic tests [18]. Unfortunately, given the structural and logistical complications that involves the viral isolation, molecular biology techniques have become a better option for the viral diagnosis. Although the use of these techniques could be very useful, the detection of the virus in serum samples is not as sensitive as it is expected, and thus the use of other samples such as the ear notch biopsies have been used due to the presence of higher amounts of virus in this area [19].

In this study, it was found the presence of PI in seronegative animals by ELISA. In order to find the segment of the genome, RT-PCR technique was done using ear cartilage samples. It was also shown the sensitivity of the technique, which allows the identification of samples with low DNA in concentration no less than 0, 00539 pM of viral genome in the host, which could be a good strategy for seeking of PI animals that are unseen on the herds.

Even though some researchers say that “to distinguish between PI and acute infection, animals showing positive results were re-tested at intervals of at least 2 weeks. Animals that tested positive on both tests were defined as PI, whereas animals with negative results in the second test were designated as having an acute infection” [9] in this group of animals, this possibility was lower because the samples were taken from seronegative animals older than 2 years with no symptoms for more than a month .

Regarding the prevalence of PI animals in Colombia (7% of the studied population), it could be said that these values are higher compared with other countries. In the case of Latin America, PI animals in Brazil were reported between 0.09 and 0.13% [20]; and in Uruguay values of 4.1% were previously shown [21]. In other places such as Belgium, the prevalence was of the 0,32% in the PI population [22] while Houe and co-workers mentioned that the prevalence around the world is between the 0,5 and 2% several years ago [19]. Also when considering the amount of positive farms in Colombia (22%), is an important fact compared with other regions worldwide due to its higher amount of positive farms. In Peru, 4% of dairy herds were found positive, similar to the situation in Japan with 7.6% of positive farms [9, 23]. All these reports belong to cattle under 2 years old. This is different from our results that were obtained in cattle older than this age. This means that the real prevalence of PIs should be higher than we show and that this group represent the option that the virus has to keep inside the asymptomatic and older animals. These PI animal serves as a reservoir for BVDV and are a source of infection for herd mates [7].

These results should be considered by the animal health authorities in order to prevent the spread of the viral infection in the Colombian cattle and thus to improve the health conditions of the animals as well as to reduce the economic impact that this virus causes on the herds. Appropriate measures should be considered in order to counteract the infection of the BVDV not only in Colombia but also in the nearby regions [15].

By the other hand, identifying the circulating species in a specific population should be described mainly together with the vaccines programs in order to have a higher chance of a protective effect and in order to know the epidemiological distribution of the viral infection. The currently available vaccine covers BVDV-1, as it is considered the most prevalent species in the world. Nowadays, it has been described at least 25 different genotypes of BVDV-1 and BVDV-2, with the proposal of a particular geographical distribution [4]. In this study, it was reported the G1a isolated from the Colombian samples. Even if BVDV-1 has been reported as the most frequent species [7], in some other regions different species have been reported. This is the case of previous reports in Brazil [20], Italy [24], India [25], and Bangladesh [26] in which the presence of BVDV-2 and BVDV-3 were reported as the only circulating species, while studies from Uruguay, Argentina and Chile also reported BVDV-1 as a single circulating variant [21, 27, 28]. However, evidence of mixed infections has also been reported as in the case of North America where phylogenetic analyses of the 5’UTR region for 119 isolates revealed the presence of the BVDV-1 and 2 [29]. In Europe, a study performed in Germany, it was found both the BVDV-1 and 2 as well [30]. When the species were typed, BVDV-1a was found in all of them, even when the most frequent genotype is 1b [2, 4, 31, 32]. Just a few regions have this type as a predominant [33]. This means that in case that a vaccination program will be implemented, the vaccine should have, at least, both types.

This is the first report in Colombia in which the circulating genotype in the PI population is reported. These results are not only important for the epidemiological background in the country but also to have an update of the current infection of the BVDV in the herds, which should be considered by the animal health authorities as higher prevalence rates was reported compared with other regions in the world.

## Conclusion

The prevalence of PI animals in some regions of Colombian was higher than it was expected. It was found BVDV- 1a as the only genotype circulating within the country. These results should be considered to include the BVDV as a mandatory notification agent in order to decrease the impact of this viral infection in the livestock industry, which still is one of the most important industries in Colombia. An eradication program of the virus is also suggested for the future.

## Methods

### Samples collection

The samples were obtained from 5 different regions of Colombia (Caquetá, Antioquia, Cauca, Huila and Sucre) including both dairy and beef herds as well as dual purpose. A total of 45 farms located in these regions were included in the study. Sample size was determined using the WinEpiscope platform [31] with the algorithm for estimating a percentage. A 4% prevalence of PI animals was considered [34], with a population of 22,574,780 animals in Colombia (2014 national census report) and a confidence level of 99% with 3% of accepted error. A sample size of 260 animals was considered, and only seronegative animals were included in the study. In order to determine the presence of the virus, ear cartilage was collected from each adult cow (older than 2 years of age and with previous pregnancies). After the ear cartilage sampling the animals keep their normal life.

### BVDV antibodies’ detection

As seronegative animals were needed for this study, blood samples were taken first in order to determine the presence of antibodies against the virus using a competitive ELISA kit (INGENESA ®, Spain) approved by the OIE as a diagnostic test. Positive cattle were identified following the manufacturer’s instructions and were not included for the cartilage sample.

### Detection of BVDV-persistent infected animals

After serological tests were completed, 260 seronegative cattle were selected for the detection of PI animals. Ear notch biopsies were collected and frozen at − 80 °C until used. About 1 cm3 of tissue was then fragmented in 1 mL of sterile PBS and centrifuged at 13.000×g for 5 min. RNA was extracted from the supernatant using a RNeasy Mini Kit (QIAGEN®, Germany) and stored at − 80 °C until used. Two steps RT-PCR was performed for the detection of the virus, in which the retrotranscription step was carried out with random primers, (Invitrogen® USA), MLV-RT (Invitrogen®, USA), dNTPs (Invitrogen®, USA) and RNA out (Invitrogen®, USA). After this, it was used the cDNA for the PCR using primers previously described [35] directed to the 5’UTR of the virus with an expected product of 288 bp with a PCR master mix (Roche ®). The sensitivity of the PCR was tested by cloning the PCR product on a pELMO vector (See section below) [36]. The results were observed on 1.5% agarose gels prepared in 1× TAE (Biorad®, USA) and dyed with 1× HydraGreen fluorescent inter-calating dye (ACTGene, USA). DNA extracted from a blood sample of an infected animal was used as positive control for BVDV and RNase- and DNase-free water as negative amplification control.

### RT-PCR sensitivity test

In order to determine the sensitivity of the RT-PCR used in this study, the purified PCR product was cloned in the pELMO vector [36]. After the PCR amplification of the cDNA directed to the regions of 5’UTR, the PCR product was purified using the Wizard® Genomic DNA Purification Kit (Promega®, USA).. Each purified PCR product was then ligated with the pELMO vector with a T4 DNA ligase (Promega®, USA). Ligation reactions were incubated for 12 h at 16 °C, in accordance with the manufacturer’s recommendations. The subsequent transformation of *E. coli* TOP10 was carried out with 100 μL *E. coli* TOP10 chemically competent cells (Invitrogen®, USA), which facilitates the cloning conditions of the plasmid and consequent selection of the successful clones with ampicillin. Bacterial cells were incubated with 5 μL of the ligation product during 20 min in ice, with a further incubation in heat at 42 °C for 30 s and finally cultured in SOC medium at 37 °C shaking for 1 h. Afterwards, the transformed cells were grown in Luria-Bertani agar plates (LB) containing 100 μg/mL ampicillin. Transformed cells were verified using colony PCR after 16 h of growth. Colony PCR was performed using directly individual transformed cells placed on the mixture of the PCR including the specific primers of the region cloned in the plasmid, in order to guarantee that the recombinant colony has the plasmid indeed, which were then visualized in 2% agarose gels by horizontal electrophoresis.

The recombinant clones were cultured to purify the plasmid using a Zyppy ™ Plasmid MiniPrep kit (ZYMO RESEARCH®). Purified plasmid DNA was confirmed by sequencing and was used as a template for the sensitivity tests for the PCR. Thus, 10-base serial dilutions of the purified plasmid (126.8 ng/μL) were done, until a final concentration of 10^− 5^ ng/ μL to determine the detection limit of the PCR.

### Sequencing, phylogenetic analysis and genotyping

Virus-positive PCR products were purified from agarose gels using the High Pure PCR Product Purification Kit (Roche®, USA). Concentration of the purified product was verified with a second agarose gel after purification, loading only 1 μL of the final product.

PCR-purified products were sequenced by the Sanger technology with both forward and reverse primers in Macrogen Inc. (Seoul, Korea). Sequences obtained were edited by Staden Package (http://staden.sourceforge.net/), giving as a result a consensus sequence per sample. Afterward, combined-multiple alignment (automatic and manual) of all the Colombian sequences was performed using ClustalW in the Mega7 program [13]. For the construction of the data set, it was used the full genome sequences of BVDV previously reported on the GenBank and the Colombian isolates obtained in this study, for a total of 26 sequences with a length of 325 bp each. Data set for phylogenetic reconstruction was obtained and the General Time Reversible (GTR) evolutionary model was used with a gamma distribution regarding Akaike information criteria (AIC). Maximum likelihood phylogenetic trees were constructed also in MEGA7 software, taking into account gaps with a bootstrap model of 100 replicates for the reliability of the trees. The edition of the trees was performed using the FigTree software version 1.4.1 (Available in http://tree.bio.ed.ac.uk/software/figtree/) and a likelihood mapping was done in the Tree-Puzzle software v.5.2 [37].

## Abbreviations

5’UTR: Untranslated region
BVDV: Bovine viral diarrhea virus
BVDV-1: Bovine viral diarrhea virus genotype 1
BVDV-2: Bovine viral diarrhea virus genotype 2
BVDV-3: Bovine viral diarrhea virus genotype 3
CP: Cytopathic biotype
MCMC: Markov chain Monte Carlo
ML: Maximum likelihood
NCP: Non-cytopathic biotypes
PCR: Polymerase chain reaction
PI: Persistently infected
RT-PCR: Reverse transcription polymerase chain reaction
